# Visceral Adipose Tissue E2F1-miRNA206/210 Pathway Associates with Type 2 Diabetes in Humans with Extreme Obesity

**DOI:** 10.3390/cells11193046

**Published:** 2022-09-29

**Authors:** Nitzan Maixner, Yulia Haim, Matthias Blüher, Vered Chalifa-Caspi, Isana Veksler-Lublinsky, Nataly Makarenkov, Uri Yoel, Nava Bashan, Idit F. Liberty, Ivan Kukeev, Oleg Dukhno, Dan Levy, Assaf Rudich

**Affiliations:** 1 Department of Clinical Biochemistry and Pharmacology, Faculty of Health Sciences, Ben-Gurion University of the Negev, Beer-Sheva 84103, Israel; 2 Helmholtz Institute for Metabolic, Obesity and Vascular Research (HI-MAG) of the Helmholtz Zentrum München at the University of Leipzig and University Hospital Leipzig, 04103 Leipzig, Germany; 3 Ilse Katz Institute for Nanoscale Science & Technology, Ben-Gurion University of the Negev, Beer-Sheva 84105, Israel; 4 Department of Software & Information Systems Engineering, Faculty of Engineering, Ben-Gurion University of the Negev, Beer-Sheva 84103, Israel; 5 Diabetes, Endocrinology and Bariatric services, Soroka University Medical Center, Beer-Sheva 84105, Israel; 6 National Institute of Biotechnology in the Negev, Ben-Gurion University of the Negev, Beer-Sheva 84103, Israel; 7 The Shraga Segal Department of Microbiology and Immunology, Faculty of Health Sciences, Ben-Gurion University of the Negev, Beer-Sheva 84103, Israel

**Keywords:** obesity, E2F1, miR-206, miR-210-5p, dys-glycemia

## Abstract

*Objective:* Up-regulated expression of transcription-factor E2F1 in human visceral adipose tissue (VAT) characterizes a dysmetabolic obesity sub-phenotype. An E2F1-miRNA network has been described in multiple cancers. Here we investigated whether elevated VAT-E2F1 in obesity is associated with VAT-miRNA alterations similar to, or distinct from, those described in cancer. Furthermore, we assessed if E2F1-associated miRNA changes may contribute to the link between high- VAT-E2F1 and a dysmetabolic obesity phenotype. *Methods:* We assembled a cohort of patients with obesity and high-VAT-E2F1, matched by age, sex, ±BMI to patients with low-VAT-E2F1, with and without obesity (8 patients/groupX3 groups). We performed Nanostring©-based miRNA profiling of VAT samples from all 24 patients. Candidate E2F1-related miRNAs were validated by qPCR in an independent cohort of patients with extreme obesity, with or without type-2-diabetes (T2DM) (n = 20). Bioinformatic tools and manipulation of E2F1 expression in cells were used to establish the plausibility of the functional VAT-E2F1-miRNA network in obesity. *Results:* Among n = 798 identified miRNAs, 17 were differentially expressed in relation to E2F1 and not to obesity itself. No evidence for the cancer-related E2F1-miRNA network was identified in human VAT in obesity. In HEK293-cells, overexpression/downregulation of E2F1 correspondingly altered the expression of miRNA-206 and miRNA-210-5p, two miRNAs with reported metabolic functions consistent with those of E2F1. In VAT from both cohorts, the expression of both miRNA-206 and 210-5p intercorrelated, and correlated with the expression of E2F1. In cohort 1 we did not detect significant associations with biochemical parameters. In cohort 2 of patients with extreme obesity, all those with high VAT-E2F1 showed a diabetes-complicated obesity phenotype and higher expression of miRNA-206 and miRNA-210-5p, which also correlated with fasting glucose levels (both miRNAs) and fasting insulin (miRNA-210-5p). *Conclusions:* Whilst the previously described cancer-related E2F1-miRNA network does not appear to operate in VAT in obesity, miRNAs-206 and 210-5p may link high-E2F1 expression in VAT with diabetes-complicated extreme obesity phenotype.

## 1. Introduction

Obesity, the excessive fat accumulation that impinges on health, is a highly heterogeneous disease, presenting itself differently in patients with similar degrees of adiposity as reflected by body mass index (BMI) [1,2]. It is now well-established that this clinical heterogeneity is paralleled to, and may be attributed to, the different degrees of metabolic dysfunction that adipose tissue develops in response to excess fat accumulation [2,3,4,5,6]. The molecular basis of adipose tissue dysfunction includes pathways involved in the initiation and maintenance of chronic low-grade inflammation [7,8], molecular stress-pathway activation [9,10,11,12,13], and altered gene expression governed by transcription factors, the miRNA landscape, and epigenetic changes [14,15,16,17]. The motivation for exploring these molecular alterations of adipose tissue in a metabolically dysfunctional obesity phenotype is in the hope of identifying both markers and drivers that would inspire better obesity subtype classification and eventual development of more personalized therapeutics.

Over recent years, E2F1 has been identified as a molecular hub regulating stress and inflammatory pathways within and around adipocytes in both human and murine adipose tissue [11,12,18]. In obesity, E2F1 is elevated in the adipocyte cell fraction of visceral adipose tissue (VAT) [12]. Traditionally studied as a cell-cycle progression regulator, in the largely quiescent adipocytes, this transcription factor appears to play different roles, driving the expression and activation of stress-activated MAPK cascades [11] and of autophagy genes [12]. In pre-adipocytes, E2F1 acts as an adipogenic stimulator [19] and negatively regulates energy expenditure and mitochondrial activity via the transcriptional regulation of mitochondrial genes [20,21]. Additionally, increased E2F1 expression in human VAT is associated with altered TNF superfamily gene expression, which maintains an inflammatory paracrine loop between adipocytes and immune cells in the tissue [18]. At the whole-body level, *E2F1* mRNA expression in human VAT correlates with insulin resistance, circulating IL-6, leptin, and FFA levels [12]. Taken together, E2F1 is an emerging contributor to the endocrine-metabolic dysfunction of adipocytes and adipose tissue.

In multiple cancers (an area in which E2F1 was most studied), E2F1 expression levels were noted to be tightly connected to the abundance of specific miRNAs. In fact, a complex co-regulatory network of E2F1 and microRNAs was described and termed the “E2F1-microRNA-cancer progression network” [22]. This network consists of E2F1 as a central hub of multiple bi-directional regulatory loops, in which E2F1 governs the transcription of specific miRNAs, and these, in turn, feed back to affect its expression, either directly or via another mediating transcription factor. In colon-cancer cells, for instance, miRNA cluster members miR-17-92, 106a-363, and 106b-25 are all transcriptionally induced by E2F1 [23,24,25,26,27]. They are also up-regulated by MYC, which is itself induced by E2F1 [27]. In turn, the three miRNA clusters inhibit E2F1’s translation directly, creating an auto-feedback regulatory loop. Another miRNA family displaying a complex interaction with E2F1 is the miRNA-449a/b/c cluster, all direct transcriptional targets of E2F1 [28]. Upon their induction, miRNA-449a/b inhibit CDK2 and CDC25A (activators of Rb-phosphorylation), thereby inhibiting E2F1 release and activation [29]. Similar regulatory loops were also described between E2F1 and miRNAs-34a, -15, -16, -205, and others, thoroughly reviewed in [22]. These molecular interactions comprise intricate control over E2F1’s expression and activity in the malignant context. The existence of these interactions in other biological contexts, such as the adipose tissue in obesity, was not examined so far. This is despite an expanding body of literature implicating adipose tissue miRNAs in regulating adipose tissue and whole-body metabolic regulation [30,31].

In light of the above, we hypothesized that in human obesity adipose tissue harbors a functional E2F1-miRNA network similar to (or, alternatively, distinct from) the previously described E2F1-miRNA-cancer progression network. Up-regulated miRNAs in human VAT in obesity may act as mediators of E2F1, linking high E2F1 with its related dysmetabolic obesity phenotype.

## 2. Methods

### 2.1. Human Cohorts and VAT Samples

Participants were recruited from two independent adipose tissue bio-banks, in Beer-Sheva, Israel and in Leipzig, Germany. As described in previous publications [12,18], the two bio-banks used a coordinated methodology for adipose tissue collection, samples’ processing, and storage. Ethical approval of the study procedures was obtained by the ethics committees of the 2 centers: Ethics Committee of the University of Leipzig, approval no: 159-12-21052012, and Helsinki Ethics Committee of Soroka University Medical Center, approval no: 15-0348. All participants signed a written informed consent form after being explained the study’s procedures. Participants (18–75 years old) were recruited, before undergoing elective abdominal surgeries (bariatric or other elective procedures). Following overnight fasting, blood samples were drawn and analyzed by the clinical biochemistry and endocrinology labs for routine parameters. Visceral adipose tissue biopsies were obtained from the greater momentum during surgery and immediately delivered to the laboratory, where they were snap–frozen using liquid nitrogen, and then processed for miRNA/mRNA/protein extraction using coordinated procedures, as in [12].

### 2.2. Cohorts Assembly

Both cohorts were designed to include age sex, ±BMI -matched participants who differed in the VAT expression level of E2F1. In cohort 1 the range of E2F1 protein content in VAT was determined in samples of n = 67 donors of the Beer–Sheva bio-bank by western blotting, as previously described in [12], using anti-E2F1-mAb (GenTex, GTX-70154, Irvine, CA, USA). High-E2F1 expression was defined as the expression level in the upper 40% (i.e., 2 quintiles) of the E2F1 expression range, and low-E2F1 expression in the lower 40% (2 quintiles).

Donors in the middle quintile of VAT-E2F1 expression level were excluded to minimize misclassification bias. From the patients with high and low VAT-E2F1 expression range, we matched triplets of patients for age, sex, and BMI. We assembled triplets, each including a patient without obesity and with low E2F1, a patient with obesity and low E2F1, and a participant with obesity and high E2F1. Of note, samples of the 8 pairs from patients with obesity were already used and reported in [18] for other analyses (RNA-seq). The clinical characteristics of Cohort 1 participants are presented in Table 1.

Cohort 2 consisted of patients with extreme obesity, without or with T2DM. The range of E2F1 mRNA levels was determined by quantitative rtPCR, as follows: out of a previously described cohort of 438 adipose tissue donors [12], we selected ten age, sex, and BMI-matched pairs from the highest and lowest *E2F1* mRNA expression quintile. All patients were with extreme obesity (i.e., BMI ≥ 45 kg/m^2^). RNA from VAT samples was extracted using RNeasy Lipid tissue Mini Kit (74804, Qiagen, Hilden, Germany). Quantity and integrity of RNA were monitored with NanoVue plus Spectrophotometer (GE Healthcare, Freiburg, Germany). An amount of 1 µg of total RNA was reverse-transcribed with a high-capacity cDNA reverse transcriptase kit (4374966, Life Technologies, Darmstadt, Germany). cDNA was then processed for TaqMan probe-based quantitative real-time polymerase chain reaction (qPCR) using the QuantStudio 6 Flex Real-Time PCR System (Life Technologies, Darmstadt, Germany). The expression of *E2F1* was calculated by the standard curve method and normalized to the expression of hypoxanthine-guanine phosphoribosyltransferase 1 (*HPRT1*) as a housekeeping gene. The clinical characteristics of Cohort 2 participants are presented in Table 2.

### 2.3. miRNA Extraction and Quantification and Nanostring©

Adipose tissue samples were lysed using the OMNI TH tissue homogenizer (OMNI international) and QIAzol lysis reagent (Qiagen, 79306). Total RNA was extracted using miRNeasy mini kit (Qiagen, 217004). The extracted amount was quantified using nanodrop©. An amount of 2 µg of total RNA per sample was reverse-transcribed with TaqMan Advanced miRNA cDNA synthesis kit (Life Technologies, A28007). TaqMan system (Life Technologies, 4369016) including specific TaqMan assays for miR-206 (477968_mir), miR-210-5p (478765_mir), and miR-let-7b (478576_mir), (Life Technologies) was used for real-time PCR amplification. Relative miRNA expression was obtained after normalization to an endogenous control (miR-let-7b), using the formula 2^−∆∆Ct^. Unbiased miRNA screening in adipose tissue was performed using the Nanostring Ncounter© technology, with the help of Agentek 2019©, Yakum, Israel. In the Nanostring run, the samples were randomly divided among 2 chips. miRNA data were analyzed in Partek Genomics Suite. Only miRNAs designated as “Endogenous1” were used. Raw signals were transformed by log2 and submitted to quantile normalization. Principal Component Analysis (PCA) and sources of variation analysis showed a clear batch effect caused by the chip and a minor batch effect by the match group. To identify differentially expressed miRNAs, a 3-way ANOVA was performed per each miRNA, with the patient group as a fixed effect and two random (batch) effects: chip and match group. The ANOVA model included the following contrasts: obese E2F1-high vs. obese E2F1-low, obese E2F1-low vs. lean E2F1-low, and obese E2F1-high vs. lean E2F1-low. The *p*-values were adjusted for multiple testing using the FDR method; however, no miRNA passed the *p* = 0.05 cutoff in any of the contrasts. Therefore, given that nanostring© analysis provided merely the baseline for further molecular experimental approaches, we enabled permissive statistical cutoffs. In all subsequent analyses, nominal *p*-values were used with a 0.05 cutoff, and a fold change cutoff of 1.25. The fold of change values was written in a linear scale (minus sign indicating down-regulation).

### 2.4. Cellular Studies

Human embryonic kidney (HEK)-293 cells (ATCC, Manassas, VA, USA) were grown in DMEM containing 4.5 mM glucose, 10% FBS, 2 mM L-glutamin, and 100 U/mL penicillin-streptomycin. The medium was changed every other day. Cells were grown to 80% confluence, then transfected with siRNA/plasmid and subjected to total RNA extraction as detailed below. For miRNA quantification following manipulations, frozen cells were scraped on ice with QIAzol lysis reagent (Qiagen, 79306) and the total RNA was extracted and miRNA was quantified using similar procedures as described for adipose tissue samples. To overexpress or knockdown E2F1, cells were seeded in 6 well plates in 1 mL/well of DMEM 10% FCS and grown to 80% confluence. To overexpress E2F1, the medium was changed to fresh medium, 1 mL/well, and cells were transfected with 200 ng/well of pCMV-E2F1 or empty plasmid, using jetPRIME reagent (Poly-plus transfection Inc., New York, NY, USA), according to the manufacturer’s instructions. To knock-down E2F1, small interfering RNA(siRNA) directed against E2F1, or a non-specific (ns) control (negative control) siRNAs, ON-TARGETplus smart pools were obtained from Dharmacon (Thermo Fisher Scientific Inc., Waltham, MA, USA). Cells were transfected with 4 ng/well siRNA using jetPRIME reagent. After transfection with either plasmid or siRNA, cells were incubated for 4 h, washed, and incubated for an additional 44 h with fresh media. After a total of 48 h, cells were snap-frozen and processed for miRNA determination as described above.

### 2.5. E2F1 Chip-Seq Data Collection from Public Databases

The information for the E2F1 binding site was obtained from Chip-Atlas [32]—an integrative database covering all public Chip-seq data submitted to the NCBI SRA. The following data sets were used: Mammary gland (GSM1526876), Mesenchymal Stem Cells (GSM2046834), Hela (GSM558469), SK-MEL-147 (GSM1665998), U-266 (GSM2132555), RAJI (GSM1976292), MCF-7 (GSM699985), MDA-MB-231 (GSM2501567), and K562 cells (GSM2827553). IGV browser version 2.14.0 was used for visualization [33].

### 2.6. Statistical Analyses

The T-test/Mann-Whitney test for a 2-group comparison and Pearson’s correlation test for correlation analysis were conducted using Graphpad-Prism 9.1.0. To enable the combined analyses of the two cohorts (cohort 1-Beer-Sheva, Israel; cohort 2-Leipzig, Germany) despite operator-dependent differences between cohorts, data were presented as fold-change of the mean. Quantitative variables (expression levels, FPG, FPI) were handled as continuous variables. Missing values were excluded from the analysis and not extrapolated.

## 3. Results

### 3.1. E2F1-miRNA Associations in VAT Are Distinct from the E2F1-miRNA Cancer-Associated Co-Regulation Network

**Figure 1:**
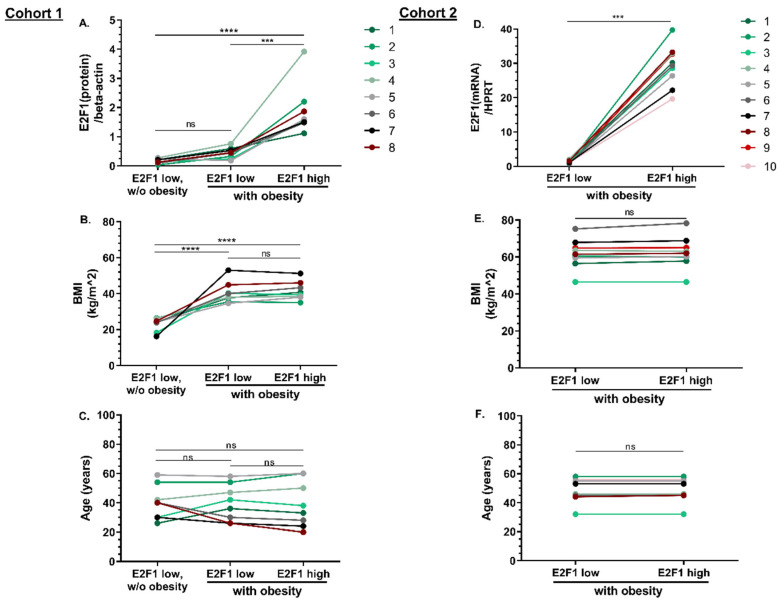
Two cohorts of matched patients with high- vs. low-E2F1 levels in VAT. Cohort 1: Human adipose tissue E2F1 protein expression range was determined on a pre-screened cohort of n = 67 persons, and high and low expression were those in the upper and lower two quintiles (40%), respectively. Based on this definition, cohort 1 (Beer-Sheva, Israel), consisted of age, sex, and (when relevant) BMI-matched triplets, each consisting of patients “without obesity and with low-E2F1 expression”, “with obesity and low-E2F1 expression” and “with obesity and high-E2F1 expression”. The matching of eight triplets of patients (i.e., n = 8 × 3 = 24) for E2F1 expression (**A**), BMI (**B**), and age (**C**) is shown. Cohort 2 (Leipzig, Germany) consisted of 10 similarly matched patients, with the following differences: E2F1 expression definition and matching; (**D**) was based on mRNA expression, and the cohort included pairs only of patients with extreme obesity (BMI ≥ 45 kg/m^2^) also matched for BMI (**E**) and age (**F**). Different colors represent each matched couple/triplet. Dots represent individual patients. *** = Pv < 0.001, **** = Pv < 0.0001, ns = non-significant.

To search for E2F1-associated VAT miRNAs, we utilized VAT samples from an assembled cohort consisting of n = 8 triplets (i.e., in total n = 24 participants, Cohort 1). To avoid any potential bias by background parameters, patients were matched from a pre-screened cohort of n = 67 for VAT-E2F1 protein level, age, sex, and BMI as follows: each triplet consisted of a patient with obesity and high VAT-E2F1 expression; an age, sex and BMI-matched participant with obesity and low VAT-E2F1 expression; and an age and sex-matched patient without obesity and with low VAT-E2F1 expression. The two matched groups of patients with obesity were previously reported [18], and more details are described in Methods (Section 2.2. *Cohorts Assembly*). Patients’ characteristics and triplet matching with the E2F1 expression, age, and BMI of Cohort 1 are shown in Table 1 and Figure 1A–C, respectively.

**Figure 2:**
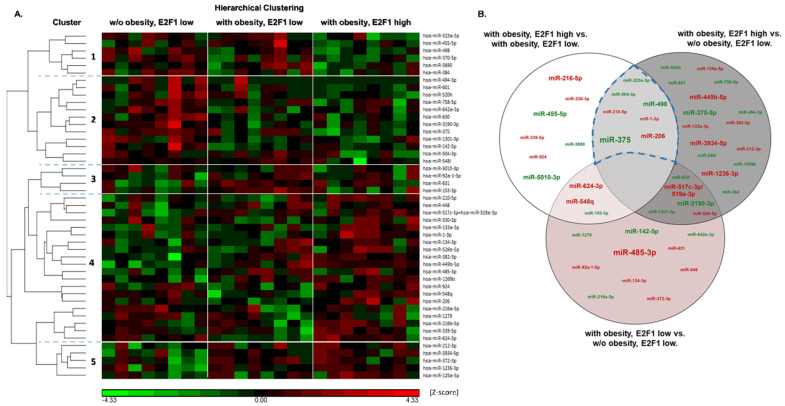
Differentially expressed miRNAs between three sub-groups of cohort 1. VAT samples of cohort 1 patients underwent miRNA extraction and quantification using Nanostring Ncounter© technology. Differentially expressed (DE) miRNAs (by at least one inter-group comparison) were hierarchically clustered and divided by trends of up/down-regulation between the three cohort groups (**A**). miRNAs differentially expressed by at least ±1.25 with Pv ≤ 0.05 were inflicted on a Venn diagram intersecting the results of three inter-group comparisons (**B**). Highlighted (dashed blue line) are miRNAs commonly DE in the two E2F1-low vs. E2F1-high comparisons. Legend: green and red fonts represent downregulated and up-regulated miRNAs, respectively. Font size is inversely proportional to Pv of differential expression for each miRNA (larger font represents lower Pv and vice versa).

miRNAs were isolated from VAT of all (i.e., 3 × 8) 24 participants, and identified using the nCounter^®^ miRNA expression panel. Nanostring© technology provides a hybridization-based, amplification-free direct count of miRNA copy numbers. Overall, 798 miRNAs were identified, out of which 47 (5.7%) were differentially expressed (DE), with a fold change (FC) > 1.25 or <−1.25, *p* < 0.05, in at least one inter-group comparison (Figure 2). Notably, a heatmap of miRNA expression profiles uncovers miRNAs primarily altered by obesity status (clusters 2 and 5, Figure 2A), being down- and up-regulated, respectively, in patients with obesity compared to those without, regardless of VAT E2F1 expression level. In contrast, other clusters of altered miRNAs exhibited differences primarily associated with E2F1 expression (clusters 1, 3, and 4, Figure 2A). We focused on the seven overlapping VAT miRNAs between the two high/low E2F1 group comparisons, to reflect those that mainly corresponded to differences in E2F1 expression irrespective of obesity (represented by the indicated area in the Venn diagram, Figure 2B). These seven miRNAs included miR-210-5p, miR-206, and its cluster-mate miRNA-1-3p, which were significantly up-regulated in high- versus low- VAT E2F1 samples, while miRNA-375, miRNA-498, miRNA-504-3p, and miRNA-323a-3p were downregulated.

**Figure 3:**
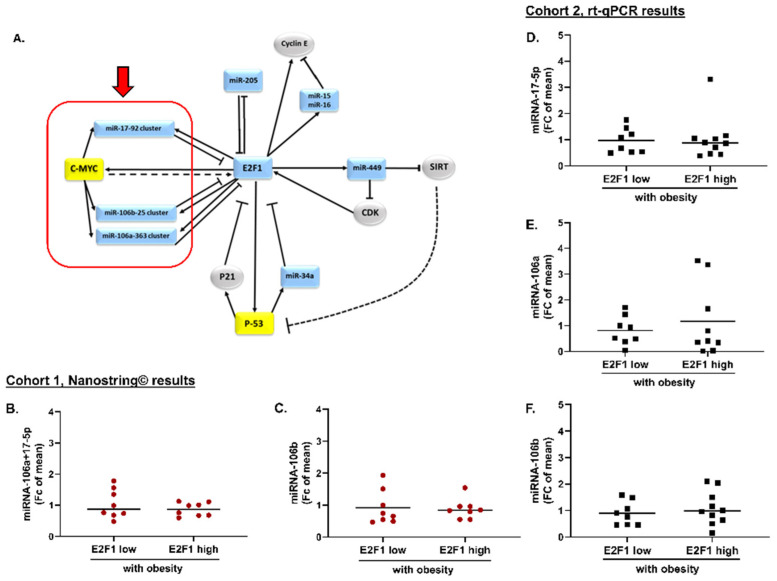
Members of the cancer-associated miRNA-E2F1 co-regulatory network are not altered in association with E2F1 in human VAT: (**A**) Illustration of the cancer-related miRNA-E2F1 co-regulation network. Expression level of miRNA106a-17-5p (**B**) and miRNA-106b (**C**) derived from nanostring© results of cohort 1, comparing patients with obesity and low- vs. high-E2F1 expression. (**D**–**F**) Quantitative rt-PCR results of VAT expression of miRNA-17-5p, miRNA-106a, and miRNA-106b, respectively, comparing patients with obesity and low- vs. high-E2F1 expression from cohort 2. Mean expressions were compared using Mann-Whitney non-parametric test. Circles and squares represent individual values, horizontal lines represent mean. All comparisons were statistically non-significant (Pv > 0.05).

Overall, members of the cancer progression E2F1–miRNA network (miRNA-17-5p and 106a/b clusters, miRNA-15a/b, miRNA-16, miRNA-34a, miRNA-205, and miRNAs-449a/b/c, Figure 3A) were absent from the identified set of VAT miRNAs altered according to E2F1 expression. (Of note, miRNA-449b-5p was elevated in high VAT-E2F1 with obesity as compared to low VAT-E2F1, without obesity. However, this miRNA failed to differentiate between low vs. high VAT-E2F1 among patients with obesity and therefore was not further considered).

### 3.2. E2F1-Associated Changes in miRNA Expression Are Validated in an Independent Patient Population with Extreme Obesity

To validate the result described above in patients with extreme obesity and either low or high E2F1 expression, we utilized VAT samples from an independent cohort, also age, sex and BMI-matched (Cohort 2, n = 20, Figure 1D–F). All participants were with extreme obesity (BMI > 45 kg/m^2^), older than patients in Cohort 1, and, remarkably, all those with low VAT-E2F1 were without T2DM and all those with high VAT-E2F1 were with T2DM, reflecting the metabolic impact associated with high-VAT-E2F1 expression [12] (Table 2). The expression of selected E2F1-associated miRNAs in VAT was assessed by real-time PCR and compared to nanostring results (Figure 3B,C). Consistent with cohort 1, no significant changes in expression levels of the cancer progression E2F1-miRNA network members miRNA-17-5p, miRNA-106a, miRNA-106b were seen between the groups (Figure 3D–F). These results suggest that in VAT of patients with extreme obesity and with T2DM associated with high VAT-E2F1 expression, up-regulation of E2F1 associates with changes in miRNA expression distinct from those known in malignant disease contexts. 

**Figure 4:**
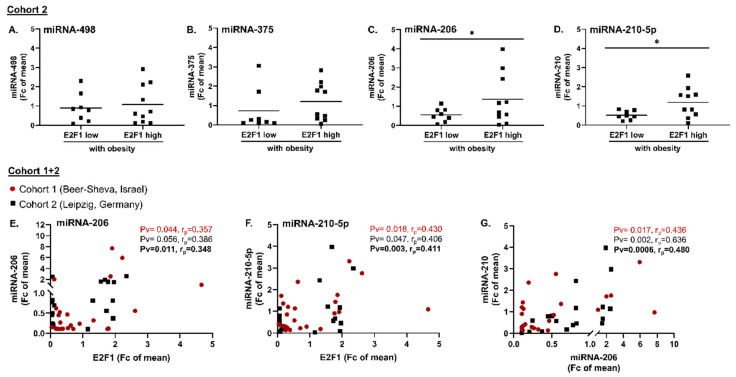
miRNA-206 and miRNA-210-5p intercorrelate and are candidate E2F1 targets. (**A**–**D**): Validation of E2F1-related DE miRNAs in human VAT using cohort 2; VAT samples of cohort 2 patients were subjected to miRNA extraction and reverse transcription as described. Expression levels of two down-regulated miRNAs in cohort 1–miR-498 (**A**) and miR-375 (**B**), and two up-regulated miRNAs–miRNA-206 (**C**) and miRNA-210-5p (**D**) were quantified using RT-qPCR. Comparison of mean miRNA expression in patients with obesity and either low- or high-E2F1 expression was performed using Mann-Whitney non-parametric test. * = Pv < 0.05. (**E**–**G**): Inter-correlations of E2F1, miRNA-206, and miRNA-210-5p; miRNA-206 (**E**) and miRNA-210-5p (**F**) correlation with adjusted E2F1 expression in human VAT. Intercorrelation between the two miRNAs (**G**). Shown are data from the two cohorts–cohort 1 participants are depicted in red circles, cohort 2 in black squares. Correlation coefficients (r_p_) and statistical significance (Pv) for each cohort separately (Cohort 1, Beer-Sheva, Israel in red; Cohort 2, Leipzig, Germany in black) and for the joint cohort (in bold black) were analyzed using Pearson’s correlation test.

We next aimed to validate E2F1-associated changes in miRNA in the VAT of patients with obesity using cohort 2. miRNA-498 and 375, down-regulated in high-E2F1 VAT in cohort 1 (Figure 2), exhibited either no change (Figure 4A) or a non-significant trend for up-regulation (Figure 4B), respectively, in cohort 2. Yet, the expression of both miRNA-206 and 210-5p was higher in VAT samples with high E2F1 expression compared to those with low E2F1 expression (Figure 4C,D, respectively), corroborating the results of cohort 1. Indeed, in both cohorts, when treated as continuous variables, the expression of miRNA-206 and 210-5p similarly correlated with an adjusted E2F1 expression (miRNA-206 correlated with E2F1 non-significantly, *p* = 0.056, in the German cohort 2) (Figure 4E,F). Moreover, both cohorts exhibited a statistically significant intercorrelation between the two E2F1-associated miRNAs (Figure 4G), possibly supporting a common up-stream regulation. The statistical significance of the results is, overall, consistent between analyses on each cohort separately, and when considered jointly as a unified cohort (Figure 4E–G). We therefore focused our further investigations on these two miRNAs as putative down-stream targets of E2F1 in adipose tissue.

### 3.3. E2F1 Regulation of miRNA-206 and miRNA-210-5p Expression

**Figure 5:**
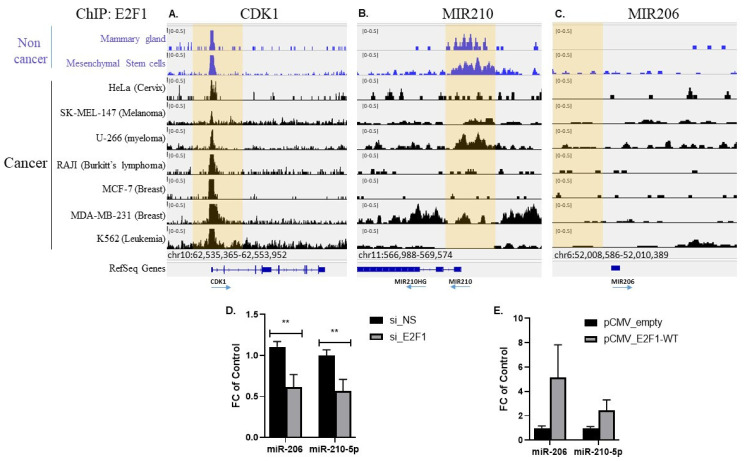
E2F1 regulates miRNA-206 and miRNA-210 expression. (**A**–**C**). E2F1 ChIP-seq results in multiple non-cancerous and cancer cells mined from publicly available databases (see Methods for detail). Depicted are genomic regions in the vicinity of CDK1–a classical E2F1 target gene (**A**), miRNA-210 (**B**), and miRNA-206 (**C**). (**D**) HEK-293 cells were grown to 80% confluence in 6 well plates and subjected to E2F1 knockdown using either non-coding siRNA (si_NS) or anti-E2F1 siRNA (si_E2F1), incubated for 48h, then snap-frozen and processed for miRNA purification. Expression levels of miRNA-206 and miRNA-210-5p were measured using qRT-PCR. Presented are results of four experiments, each with two biological replicates and two technical replicates. (**E**). Cells were transfected with plasmids encoding either “empty” pCMV plasmid or a pCMV-hE2F1 plasmid, incubated for 48h, then snap-frozen and processed for miRNA isolation. Expression levels of miRNA-206 and miRNA-210-5p were measured using qRT-PCR. Presented are results of three experiments, each with two biological replicates and two technical replicates. Results are mean ± SEM. ** = Pv ≤ 0.01. Comparison of means was done using Mann-Whitney non-parametric test. Effective E2F1 knock-down/over-expression using the exact siRNA/plasmid transfection protocol was demonstrated elsewhere (1).

To determine if E2F1 may be a direct transcriptional regulator of the two miRNAs, we first conducted a prediction analysis of putative E2F1 binding sites in the minimal promoter regions (−1000 from the transcription initiation site) of miRNA-206 and 210-5p. This DNA sequence analysis suggested a high probability for four and 10 putative binding sites for E2F1 in minimal promoter regions up-stream of miRNA-206 and 210-5p, respectively (data not shown). To gain experimental support for E2F1 binding to potential regulatory regions of the two miRNAs, we searched for publicly available E2F1 ChIP-seq databases in non-cancerous as well as in cancer cells. In all cells tested, the E2F1 ChIP signal was readily noted for the classical E2F1 target gene CDK1 (Figure 5A). For miRNA-210, the non-cancerous mammary gland and mesenchymal stem cells exhibit enrichment of E2F1 in this locus, suggesting that this miRNA may be a direct target of E2F1 (Figure 5B(. Intriguingly, among cancerous cells, the E2F1 ChIP signal was present for some cells (melanoma, myeloma, and MDA-MB-231 breast cancer cells), but not for others (HeLa, Burkitt lymphoma, MCF7 and K562) (Figure 5B). In contrast, the E2F1 ChIP signal was seemingly absent in all cell types for miRNA-206, suggesting that if regulated by E2F1, the mode of regulation is likely indirect (Figure 5C). To further support experimentally the regulation of the miR-206 and 210-5p expression by E2F1, we manipulated E2F1 expression in HEK-293 cells and tested the effect on the expression level of both miRNAs. E2F1 knock-down using siRNA (confirmed using this protocol elsewhere [18]) resulted in significant downregulation of both miRNA-206 and miRNA-210-5p (Figure 5D), while the over-expression of E2F1 resulted in a trend of up-regulation for both miRNAs (Figure 5E). Jointly, these results reinforce the hypothesis that miR-206 and miR-210-5p are up-regulated down-stream of E2F1 in VAT in human obesity.

### 3.4. miRNA-206 and miRNA-210-5p Expression in VAT May Link High VAT-E2F1 T2DM in Patients with Severe Obesity

Next, we sought to assess whether miR-206 and miR-210-5p could function as molecular mediators of E2F1, linking high-VAT expression of E2F1 with a dysmetabolic phenotype of obesity. We first examined whether the combination of miR-206 and 210-5p had any known associations with obesity-related disease. Using the miEAA online tool, we discovered that the miRNA-206/miRNA-210-5p “duo” was over-represented in the scientific literature in relation to a set of diseases associated with obesity, including, among others, lipid disorders, elevated glucose levels and diabetes complications, non-alcoholic fatty liver disease, as well as vascular calcification and atherosclerosis, all evolving from disrupted metabolic function. To gain further insight on possible mechanisms for the involvement of miR-206 and miR-210-5p in metabolic disease, we assessed their association with participants’ metabolic markers, specifically circulating lipids and blood glucose levels. To be comparable among the two cohorts, we included only patients with obesity in cohort 1. We did not detect significant associations between the two E2F1-related miRNAs and glycemic or lipid parameters in cohort 1 (Table 3).

**Figure 6:**
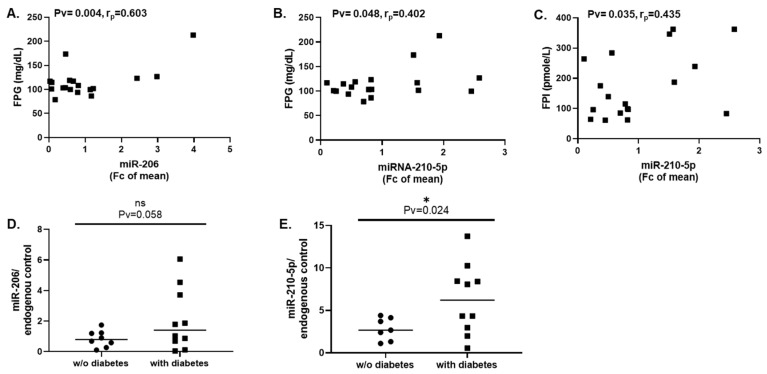
Expression of miRNA-206 and miRNA-210-5p in human VAT associates with T2DM in extreme obesity. (**A**–**C**). Correlation between expression levels of miRNA-206 and miRNA-210-5p (by rt-PCR, cohort 2) with fasting plasma glucose (FPG) or fasting plasma insulin (FPI). Correlations were performed using Pearson’s test. (**D**,**E**). Group comparison of Cohort 2: participants were divided by diabetes status (w/o T2DM = low VAT-E2F1; with T2DM = high VAT-E2F1), and mean expression levels of miRNA-206 and 210-5p were compared between the two groups using Mann-Whitney non-parametric test. * = Pv < 0.05, ns = non-significant.

Yet, in cohort 2, in patients with extreme obesity and in whom high-VAT-E2F1 was associated with T2DM, while lipid profile was not associated with miRNA-206 or miRNA-210-5p expression (Table 4), significant correlations were observed between the miRNAs and glycemic parameters (FPG, FPI): miRNA-206 correlated with FPG levels and miR-210-5p correlated with both FPG and fasting plasma insulin (FPI) levels (Table 4, Figure 6A–C, respectively), signifying a systemic glucose-intolerant/insulin-resistant profile. In accordance, by group analysis, those with high-VAT-E2F1 and T2DM exhibited a significantly higher average of miR-210-5p levels (Pv = 0.024), and a nearly significant trend for higher miR-206 levels (Pv = 0.058) (Figure 6D,E).

## 4. Discussion

E2F1 is a well-studied transcription factor that has been shown to be central in multiple biological networks. E2F1-mediated regulation of gene expression involves several molecular mechanisms, including direct binding to target gene promoters, resulting in transcriptional activation/repression and indirect mechanisms such as via miRNAs. Mostly studied in the context of cell-cycle regulation, a cancer-related E2F1-miRNA network has been documented [22]. In it, E2F1 regulates the transcription of certain miRNAs, which in turn regulates their targets, E2F1 itself being one. Beyond its roles in cell-cycle control, E2F1’s involvement in regulating metabolism has been studied in key metabolic organs including the pancreatic beta cells [19,34], liver [35,36,37,38], and adipose tissue. In the latter, our previous studies placed E2F1 at a central hub within a molecular network, regulating adipocyte autophagy, MAPK stress-signaling, and gene expression of TNF superfamily members [11,12,18]. Each of these down-stream effectors of E2F1 within adipose tissue only partially mediates E2F1’s putative role in linking obesity with a dysmetabolic obesity phenotype. In the present study, we demonstrate, using two independent cohorts, that in adipose tissue in obesity, an E2F1-miRNA network distinct from that shown in cancer may operate. miRNA-206 and miRNA-210-5p were elevated in VAT of patients with obesity in association with E2F1, and were not altered between patients without vs. with obesity but with comparable E2F1 levels. In two separate populations, one of which (cohort 2) included patients with extreme obesity, in which high VAT-E2F1 corresponded to obesity complicated by T2DM, expression levels of both miRNAs intercorrelated, each also with E2F1 expression itself. Indeed, the possibility of direct (for miRNA-210-5p) or indirect (for miRNA-206) regulation by E2F1 in different cell types was proposed by mining deposited E2F1 ChIP datasets, and by experimentally demonstrating that siRNA-mediated E2F1 knockdown significantly decreased expression, and E2F1 over-expression trended to over-express both miRNA-206 and 210-5p. Finally, consistent with scientific literature linking these miRNAs with a range of metabolic diseases, we observed in patients with extreme obesity, that VAT expression of these miRNAs correlated with T2DM. Overall, our data suggest that E2F1 up-regulates the expression of miRNA-206 and 210-5p in human VAT, situating the E2F1-miRNA-206/210-5p pathway as an addition to an intricate E2F1-regulated network in visceral adipose tissue, which may contribute to an obesity phenotype complicated by T2DM. 

Although, to the best of our knowledge, the E2F1-mediated regulation of miRNA-206 and miRNA-210-5p is a novel finding, our results are supported by substantial literature on the physiological functions of both miRNAs, which seem to substantially overlap with those of E2F1. In particular, miRNA-210 has been reported to function as a major “hypoxiamiR” [39], hypoxia-inducible miRNA in a wide range of cells, serving to adjust the cells to hypoxic conditions. In this context, miRNA-210 was shown to alter cellular metabolism by compromising mitochondrial function and limiting glycolytic-to-mitochondrial substrate transition via targeting components of the Krebs cycle and electron transport chain [40,41,42]. Concordantly, cells transfected with miRNA-210 demonstrated decreased oxygen consumption and a greater dependence on glycolysis for energy metabolism [42]. Indeed, recent studies indicate that exosomal miRNA-210 may disrupt adipocyte glucose mitochondrial oxidation and inhibit the browning of naïve adipocytes [43,44]. Remarkably, E2F1 was repeatedly demonstrated to inhibit oxidative metabolism via inhibition of metabolic flux to the mitochondria (regulating pyruvate dehydrogenase complex), mitochondrial biogenesis and enzymatic activity [20,21,45], and was suitably demonstrated to inhibit adipocyte beiging/browning (i.e., drive adipocyte “whitening” [46]). Thus, it is plausible that miR-210 mediates E2F1-dependent adipose tissue whitening in obesity.

A similar E2F1-mediatory role for miRNA-206 can be envisioned, as miRNA-206 was shown to interfere with intracellular glucose utilization and increased E2F1 expression in human VAT associated with decreased systemic glucose infusion rate during hyperinsulinemic-euglycemic clamp studies [12]. In pancreatic beta-cells, miRNA-206 directly inhibits glucokinase(GK) activity via a conserved binding site at the 3′-UTR of this gene [47,48], thereby interfering with glucose-stimulated insulin secretion. Interestingly, miRNA-206 expression in beta-cells was shown to increase upon high-fat feeding [47]. Beyond its effect on GK, which is a beta-cell/hepatocyte-specific enzyme, miRNA-206 similarly regulates hexokinase(HK) in malignant cells [49]. miRNA-206-driven inhibition was also noted on glucose-6-phosphate dehydrogenase(G6PD) [50] and on the end-glycolytic enzyme pyruvate kinase(PK) [51], decelerating hexose monophosphate shunt and glycolytic flux, and thereby, glucose handling. Indeed, several separate reports demonstrated that overexpression of miRNA-206 results in decreased glucose uptake into a wide variety of cells, and vice-versa, its elimination resulted in improved glucose handling [47,49,51]. Albeit not directly studied so far in adipose tissue, our results propose that miR-206 could similarly operate in adipocytes down-stream of E2F1, acting as a mediator of E2F1-associated dysglycemia.

As mentioned above, a growing body of literature implicates E2F1 in the regulation of metabolism in key metabolic organs: by up-regulating PDH kinase 4(PDK4), which phosphorylates and inhibits the PDH complex, E2F1 supports glycolysis over glucose oxidation in myocytes [45]. In hepatocytes, up-regulated E2F1 drives lipid accumulation and gluconeogenesis, thus, promoting hepatic steatosis and systemic hyperglycemia [35,36,37,38]. In pancreatic β-cells, it regulates the expression of *Kir6.2*, driving glucose-stimulated insulin secretion and supporting the hyper-insulinemic response [19,34]. In the adipocyte, as mentioned, it promotes “whitening” (=inhibits beiging) [46]. In accordance, in E2F1-KO mice, despite the decreased pancreatic size (attributed to impaired postnatal growth), mice were not diabetic due to concurrent increased adipose browning and insulin sensitivity [19]. The latter phenotype is consistent with human data implicating E2F1’s role in the generation of systemic insulin resistance when up-regulated in obesity. E2F1 is also mechanistically related to inflammation: both acute inflammatory stimuli (TPA, arsenate) [52,53] and chronic inflammation [54] induce E2F1 protein and mRNA expression and activate the retinoblastoma-E2F1 axis, respectively. We have recently demonstrated that in adipose tissue, lymphocytes-derived TL1A(TNFSF15) can directly induce the adipose tissue E2F1 expression, which in turn stimulates the expression of adipose tissue TRAIL(TNFSF10) that further induces TL1A, generating a feed-forward, E2F1-inflammatory cytokine feedback loop [18]. In all of these sites and mechanisms, the respective roles of E2F1-induced miRNA-206 and 210-5p remain to be discovered, as is the potential beneficial effects of antagonizing these E2F1-effector miRNAs. Our results suggest that this approach may prove beneficial, particularly in dysglycemic persons with obesity and high-VAT-E2F1 expression.

There are a few strengths, but also limitations to our study, which warrant consideration. Paralleling the “general” reproducibility crisis in bio-medical research, studies on miRNAs have particularly seen low reproducibility among cohorts. Hence, our study is strengthened by insisting on results’ validation using two independent, carefully constructed cohorts, from two centers coordinated by the pre-analytic procedures (human tissue sampling and handling). Cohort 2 represents a more extreme obesity phenotype, with older patients in whom high VAT-E2F1 is uniformly associated with obesity being complicated by T2DM. Moreover, to ensure that results do not merely depend on specific methodological approaches, VAT-E2F1 expression was assessed at the protein level in cohort 1, and at the mRNA level in cohort 2. Similarly, the determination of miRNAs was performed using Nanostring© or rtPCR in cohorts 1 and 2, respectively. The statistical approach undertaken also allows us to carefully predict the chance that our results are random by considering each cohort separately, as well as when joined into a fused cohort. An additional strength of our study is the experimental confirmation of bioinformatic predictions, particularly the regulation of the two miRNAs by manipulating E2F1 levels. Yet, the experimental model we used for miRNA screening consisted of whole adipose tissue, while the origin of VAT-E2F1 is likely the adipocytes [12]. We therefore cannot rule out that our approach “dilutes” or masks adipocyte-specific processes. Thus, we may have missed adipocyte-only E2F1-related miRNAs consistent with the cancer-associated E2F1-miRNA network, which we initially anticipated. It is also worth noting that the mechanistic confirmation provided here for E2F1-regulated expression of miRNA-206 and miRNA-210-5p is demonstrated in HEK293 cells rather than adipocytes. This is due to the well-known difficulty of genetically manipulating mature adipocytes [55], with human mature adipocytes being an even greater challenge. Yet, HEK-293 cells are of human origin, gain- and loss-of-function approaches yielded mirroring results, and were combined with cross-sectional analyses of human adipose tissue, supporting our findings.

**Scheme 1:**
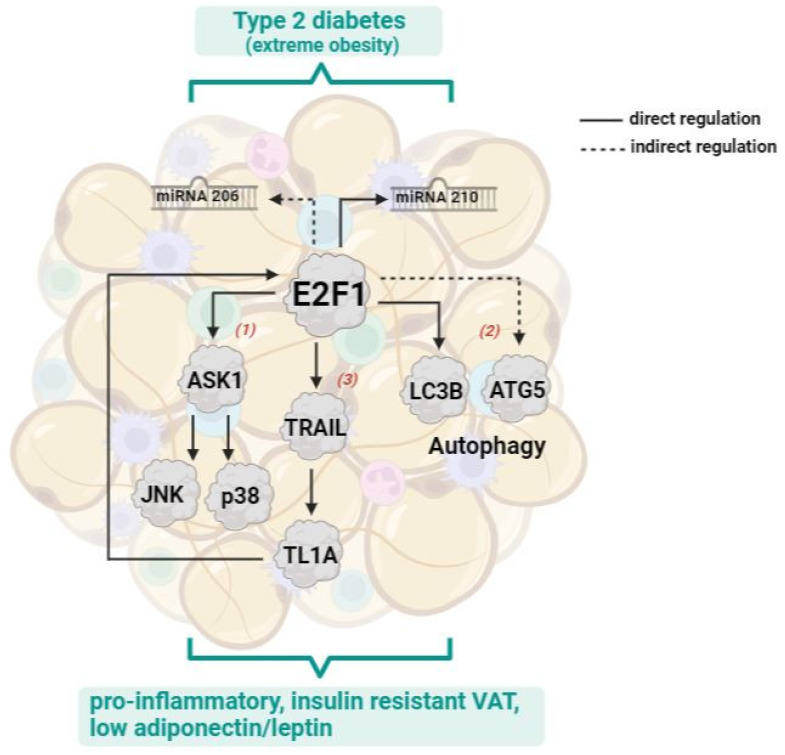
The expanded human VAT E2F1 network. In human adipose tissue in obesity, increased expression of E2F1 in VAT was shown to regulate autophagy genes [12], the MAP3K5/ASK1 MAP kinase pathway [11], and TNF superfamily members [56], linking obesity with a pro-inflammatory, insulin resistant, and dysfunctional adipose tissue obesity phenotype. The present study suggests that in addition E2F1 up-regulates the expression of miRNA206 and 210-5p, which link between extreme obesity and T2DM.

In conclusion, this study expands our understanding of the E2F1 molecular network that operates in human visceral adipose tissue in obesity, and how it associates with extreme dysmetabolic obesity complicated by T2DM (Scheme 1). Differing from our hypothesis we found no evidence for a functional E2F1-miRNA network reminiscent of that which was described in cancer (the E2F1-miRNA cancer-progression network). Yet, in human visceral adipose tissue, we proposed that in addition to E2F1-mediated regulation of ASK1 (MAP3K5) MAP kinase signaling pathway, autophagy, and TNF superfamily members, E2F1 regulates miRNA 206 and 210-5p. In light of this study’s objectives, we demonstrate that in extreme obesity setting these possibly link high-VAT-E2F1 with T2DM and systemic insulin resistance. Thus, the importance of the study is: i. expanding basic pathophysiological understanding of the molecular mediators that link high E2F1 in VAT to a metabolically complicated extreme obesity; ii. given that miRNA-based molecules are potential molecules for drug development, our findings propose miRNA-206 and 210-5p as putative new targets for alleviating obesity-associated dysglycemia.

## Figures and Tables

**Table 1:** Clinical characteristics of participants in cohort 1.

Cohort 1- Beer-Sheva, Israel				
	(Group #1) w/o Obesity	(Group #2) w/ Obesity, Low E2F1	(Group #3) w/ Obesity, High E2F1	*p*-Value
n	8	8	8	
Age (years)	40.1 ± 11	41.2 ± 12	40.2 ± 17	ns, 0.95
Sex (M:F ratio)	3:5	3:5	3:5	
BMI (kg/m^2^)	23 ± 3	40.5 ± 6	41.5 ± 5	*, #, 0.0004
FPG (mg/dL)	98.1 ± 22	105.2 ± 36	81.6 ± 11	ns, 0.2571
TG (mg/dL)	140.6 ± 77	172.6 ± 54	157.9 ± 80	ns, 0.6034
LDL (mg/dL)	101 ± 29	113.2 ± 19	119.7 ± 13	ns, 0.3491
HDL (mg/dL)	36.7 ± 17	40.7 ± 8	42.4 ± 9	ns, 0.5073
Diabetes (T2DM) (yes/no)	0/8	1/7	0/8	

*—Significant difference between groups 1 and 2, Pv < 0.05; #—Significant difference between groups 1 and 3, Pv < 0.05; ns—non significant.

**Table 2:** Clinical characteristics of participants in cohort 2.

Cohort 2- Leipzig, Germany			
	(Group #1) w/ Extreme Obesity, Low *E2F1*, w/o T2DM	(Group #2) w/ Extreme Obesity, High *E2F1*, w/ T2DM	*p*-Value
n	10	10	
Age (years)	50.2 ± 8	50.3 ± 8	ns, 0.4802
Sex (M:F ratio)	5:5	5:5	
BMI (kg/m^2^)	62.1 ± 7	62.8 ± 8	ns, 0.4631
FPG (mg/dL)	110.2 ± 9	129.3 ± 35	*, 0.0019
TG (mg/dL)	101.2 ± 30	154.7 ± 49	*, 0.0276
LDL (mg/dL)	129.5 ± 15	99.9 ± 50	ns, 0.1518
HDL (mg/dL)	52.9 ± 4	44.7 ± 23	ns, 0.0939
Diabetes (T2DM) (yes/no)	0/10	10/0	

*—Significant difference, Pv < 0.05; ns—non significant.

**Table 3:** Correlations between glycemic and circulating lipids parameters and miRNA-206 and miRNA-210-5p in cohort 1.

Cohort 1- Beer-Sheva, Israel				
	miRNA-206		miRNA-210-5p	
	P_v_	r_p_	P_v_	r_p_
FPG	0.409	0.049	0.267	−0.133
FPI	NA	NA	NA	NA
TG	0.227	−0.160	0.053	−0.338
LDL	0.208	−0.187	0.065	0.340
HDL	0.159	0.218	0.171	0.207

FPG—fasting plasma glucose; FPI—fasting plasma insulin; TG—triglycerides; LDL—low-density lipoprotein cholesterol (calculated); HDL—high-density lipoprotein cholesterol.

**Table 4:** Correlations between glycemic and circulating lipids parameters and miRNA-206 and miRNA-210-5p in cohort 2.

Cohort 2- Leipzig, Germany				
	miRNA-206		miRNA-210-5p	
	P_v_	r_p_	P_v_	r_p_
FPG	**0.004**	**0.604**	**0.048**	**0.402**
FPI	0.180	0.229	**0.035**	**0.435**
TG	0.058	0.424	0.222	0.213
LDL	0.143	−0.284	−0.214	−0.231
HDL	0.128	−0.326	0.062	−0.431

FPG—fasting plasma glucose; FPI—fasting plasma insulin; TG—triglycerides; LDL—low-density lipoprotein cholesterol (calculated); HDL—high-density lipoprotein cholesterol.

## Data Availability

All data will be made available by request to the corresponding authors.

## Abbreviations

VAT	visceral adipose tissue
BMI	body mass index
FFA	free fatty acids
FPG	fasting plasma glucose
FPI	fasting plasma insulin
DE	differentially expressed
FC	fold change
W/	with
W/O	without
CDK1	cyclin dependent kinase 1
ChIP	chromatin immunoprecipitation
